# Comparison of efficacy of intravenous versus intramuscular injection meglumine antimoniate in patients of cutaneous leishmaniasis

**DOI:** 10.12669/pjms.42.2.12520

**Published:** 2026-02

**Authors:** Najia Ahmad, Shafia Shaikh, Saima Ali Khan, Moniba Saeed

**Affiliations:** 1 Najia Ahmad Department of Dermatology, Combined Military Hospital, Quetta (CMH Qta)/Quetta Institute of Medical Sciences (QIMS), Balochistan, Pakistan; 2 Shafia Shaikh Department of Dermatology, Combined Military Hospital, Quetta (CMH Qta)/Quetta Institute of Medical Sciences (QIMS), Balochistan, Pakistan; 3 Saima Ali Khan Department of Dermatology, Combined Military Hospital, Quetta (CMH Qta)/Quetta Institute of Medical Sciences (QIMS), Balochistan, Pakistan; 4 Moniba Saeed Department of Dermatology, Combined Military Hospital, Quetta (CMH Qta)/Quetta Institute of Medical Sciences (QIMS), Balochistan, Pakistan

**Keywords:** Antimoniate, Cutaneous leishmaniasis, Efficacy, Intravenous parasitic, Meglumine intramuscular, Neglected tropical diseases, Phlebotomus, Safety, Zoonotic diseases

## Abstract

**Objective:**

To compare the efficacy of intravenous versus intramuscular meglumine antimoniate in the treatment of cutaneous leishmaniasis.

**Methodology:**

This randomized controlled trial was carried out in Dermatology Department of Tertiary Care Hospital, Quetta, from April to October 2024. Patients with biopsy proven cutaneous leishmaniasis were randomized into intravenous and intramuscular groups. The intravenous group received 15mg/kg/day of meglumine antimoniate diluted in 100 ml of 5% glucose solution over 60 minutes, while the intramuscular group received a similar dose into gluteal muscles. All patients were observed for treatment response at 15, 30, 45 and 60 days of treatment unless cured.

**Results:**

Ninety patients were divided into two groups of 45 each. Participants mean age was 30.10±5.75 years. The mean number of lesions were 4.28±3.67. The mean number of injections was 36.91±8.296. Intravenous group patients demonstrated early recovery with mean number of injections 28.78±2.57 in comparison to that in intramuscular group 39.56±2.80, this difference in efficacy was statistically significant (p<0.001). Pain at the injection site was significantly lower in the intravenous group compared to the intramuscular group (p<0.05).

**Conclusion:**

Intravenous administration of injection meglumine antimoniate is superior to Intramuscular in terms of enhanced efficacy and improved patient outcome. There were statistically insignificant side effects between two groups except for pain at injection site which was more with intramuscular route.

***Registration number:*** IRCT20210823052264N4.

## INTRODUCTION

Leishmaniasis is caused by intracellular flagellate protozoan Leishmania.^1^ It is transmitted by infected female sandfly bite, Phlebotomus (Old World) and Lutzomyia (New World). There is variable presentation of the disease ranging from simple cutaneous ulcers to fatal visceral leishmaniasis. On the basis of leishmania species, host genetic factors and immunity there are three forms of leishmaniasis: cutaneous, mucocutaneous and visceral, with cutaneous leishmaniasis (CL) being the most common form.^2^ CL may present as papule, nodule, nodular ulceration and plaque. A papule at the site of inoculation is followed by an ulcer. Ulcer is the most common presentation.^1^ The sandfly has tendency to bite the exposed body parts and the lesions heal with scaring. These scars on visibly prominent areas such as face, hands and feet can be disfiguring and cause significant psychological stress to the patient.^3^

According to World Health Organization (WHO), 2023 report approximately one million new cases of CL are reported each year across the globe, of which 95% are in America, the Mediterranean Basin, the Middle East and Central Asia.^4^ It is present in almost 100 countries, with a total 350 million risk population.^2^ In Asia, it is prevalent in Pakistan, Iran, Afghanistan, India, China and Saudia Arabia. Around 20,000 cases occur annually in Pakistan and have been extensively reported in all four provinces Sindh, Baluchistan, Punjab and Khyber Pakhtunkhwa.^3^,^4^ Directorate General of Health Services Punjab on 5th June 2023 highlighted the increasing number of CL cases.^5^ The treatment choice depends upon clinical form of disease, drugs’ efficacy, toxicity, cost of treatment, therapeutic scheme and patient acceptability.^6^ Local therapies for treating CL include physical modalities (cryotherapy, thermotherapy), topical drugs such as paromomycin and intralesional therapies (antimony and pentamidine).^7^ Systemic drugs include pentavalent antimonial (Sb^V^) compounds, azole antifungal, amphotericin B, pentamidine, miltefosine and granulocyte macrophage colony-stimulating factor.^8^ According to WHO guidelines for treatment of CL, pentavalent antimonial can be used in treatment of all types of CL after assessing the risk benefit ratio.^9^

WHO states that CL caused by Leishmania Major is self resolving with cure of 50%–75% at 4–6 month, while Lesions caused by Leishmania Tropica needs treatment because of risk of human-to-human transmission, development of drug resistance. Due to financial constraint in part of patient and non-availability of species identification facility, species analysis is not done in every case. Treatment is crucial to hasten healing, prevent scarring, and reduce relapse. The decision of treating the lesion is also dependent on its size, site, patient acceptability and co-morbidities.^5^ Meglumine antimoniate (MA) is the mainstay of treatment for CL.^2^,^8^ It can be given by both intralesional (IL) and parenteral route.^9^ It disrupts ATP production, induces oxidative stress and cause lipid peroxidation of the membrane, leading to loss of membrane integrity. It also inhibits DNA and protein synthesis in the protozoa.^10^

Systemic MA is usually given via intramuscular (IM) route, but it is a very painful injection. About 10-20ml of the drug has to be injected daily, which is quite distressing for the patient. Besides that instead of the usual 21 days therapy, the duration of treatment required for complete healing of the lesions is usually increased that may be attributed to poor tolerability of injection. We expect intravenous (IV) route to be more effective and comfortable for the patient. There are numerous comparative studies between IL and systemic MA, but no study could be found comparing IV with IM route. The rationale of this study is to compare the efficacy and safety of IV versus IM MA, that will provide a valuable insight into the duration of wound healing, patient comfort and the feasibility of each treatment method. The result of this trial would help physicians and dermatologists in making evidence-based decisions on choosing the most appropriate route of injection MA administration for the treatment of CL.

## METHODOLOGY

This randomized controlled trial was conducted in Department of Dermatology of a tertiary care hospital in Quetta. The duration of study was from April 2024 to October 2024. Approval from institutional ethical review board (CMH QTA- IERB/18/2024) was taken on 29 March 2024. IRCT registration was done on 03-04-2024 with IRCT registration number: IRCT20210823052264N4. Sample size of 90 was calculated using WHO calculator, with confidence level 95%, margin of error 5%, anticipated population proportion P1 of 94.4%^11^ and anticipated population proportion P2 of 78%.^12^ Non probability consecutive sampling was done. The study was single-blind because of patients were aware of the route of drug administration, but the assessment of lesion and data analysis was performed by an independent evaluator who was unaware of the treatment allocation, minimizing observer bias

Patients were divided into two groups: Group-A and Group-B, each with 45 patients. This random assignment was done using lottery method. Group-A was given IV and Group-B was given IM MA. Informed consent taken prior to treatment. Patients of both genders, aged 15 to 80 years, with biopsy proven CL and any of the following characteristics of lesion were included:

Lesion ≥4 cm, lesion(s) located in sites not suitable for local treatment, lesions in which scaring would be debilitating or severely disfiguring like on face, lower limb or over a joint and/or lesions involving mucosa or cartilage. Patients with lesion less than 4cm, allergy to antimonials, baseline ECG changes, patients included in other research protocols, pregnant and breastfeeding women, those who refused to accept the diagnostic procedures or had mucosal, mucocutaneous or visceral leishmaniasis were excluded. According to Manual for case management of cutaneous leishmaniasis in the WHO Eastern Mediterranean Region: “Sb5+ pharmacokinetics are almost identical by intravenous and intramuscular routes”.^5^ MA meglumine antimoniate (MA) is commercialised by Sanofi-Aventis under the trade name Glucantime. It is recommended in WHO Guidelines and is given via IV route in study by Carvelho et al. The IV group received 15mg/kg/day of MA diluted in 100ml of 5% glucose solution, given over 60 minutes with cardiac monitoring while the IM group received a similar dose as per weight into gluteal muscles. One injection is 5ml and 15ml means three injections which is the usual dose given to patient per day according to their weight. All the patients were observed for treatment response at 15, 30, 45 and 60 days of treatment unless cured. The physician assessing the response did not know to which group the patients belonged.

The results were grouped into poor response, fair response, good response and complete healing. Poor response was <50% improvement from start of treatment, fair response was 50-70% healing, good response was 76-90% healing of the lesion, while 100% resolution of induration and ulceration was defined as complete healing. Data was analyzed with IBM-SPSS V27. Chi-square test was applied to compare efficacy of both groups, taken p≤0.05 as significant. Based on factors such as age, lesion count, number of injections, drug delivery method, wound healing and potential side effects of drug therapy, stratification was performed. A chi-square test was conducted post-stratification for both groups, with significance set at p ≤ 0.05.

## RESULTS

We evaluated the efficacy and safety of IV and IM routes of administration of injection MA for treatment of CL. Ninety participants of biopsy proven leishmaniasis completed the study. All participants were male. The mean age of participants was 30.31±5.52. years. The mean age in IV group was 30.73±6.429 years, while it was 29.47±4.980 years in IM group. The mean number of lesions was 4.46±3.67. Mean number of lesions in IV group 4.78±3.87 was comparable to 4.13±3.48 in IM group.

Overall Cure was achieved in mean 36.91±8.29 days. Intravenous group patients demonstrated early recovery with mean number of days 28.78±2.57 in comparison to that in intramuscular group 39.56±2.80, irrespective of other parameters including age, gender, lesion site and type of lesion. This difference in efficacy was statistically significant (p<0.001). The comparison of both groups considering their response at 15, 30, 45 and 60 days is listed in Table-I. Both groups showed sustained response when evaluated at day 60.

**Table-I:** Treatment duration and treatment response in two study groups.

Duration of treatment			Efficacy/cure rate		p-value
			Group-A (n=45)	Group-B (n=45)	
***15 Days***					
Poor response (<50% healing)			4(8.9%)	43(95.6%)	0.00
Fair response (50-75% healing)			37(82.2%)	2(4.4%)	
Good response (75-99% healing)			4(8.9%)	0(0%)	
Complete healing)	response	(100%	0(0%)	0(0%)	
***30 Days***					
Poor response (<50% healing)			0(0%)	0(0%)	0.00
Fair response (50-75% healing)			1(2.2%)	18(40.0%)	
Good response (75-99% healing)			6(13.3%)	25(55.6%)	
Complete healing)	response	(100%	38(84.4%)	2(2.2%)	
***45 Days***					
Poor response (<50% healing)			0(0%)	0(0%)	0.012
Fair response (50-75% healing)			0(0%)	2(4.4%)	
Good response (75-99% healing)			45(100%)	6(13.3%)	
Complete healing)	response	(100%	45(100%)	37(82.2%)	
***60 Days***					
Poor response (<50% healing)			0(0%)	0(0%)	Constant
Fair response (50-75% healing)			0(0%)	0(0%)	improvement
Good response (75-99% healing)			0(0%)	0 (0%)	
Complete healing)	response	(100%	45 (100%)	45 (100%)	

**Table-II:** Adverse Effects between two study groups.

Side effects	Total	Group-A	Group-B	P-value
Headache	12(13.0%)	7(7.6%)	5(5.4%)	0.75
Injection site pain	15(16.6%)	0	15(16.6%)	0.00
Nausea/ vomiting	10(10.9%)	7(7.6%)	3(3.3%)	0.15
Arthralgia	10(10.8%)	5(5.4%)	5(5.4%)	1.0
Myalgias	12(13.2%)	6(6.6%)	6(6.6%)	1.0
ECG changes	19(21.0%)	11(12.2%)	8(8.8%)	0.6
Raised ALT	9(10%)	5(5.5%)	4(4.4%)	1.0
Eosinophilia	1(2.2%)	1(1.1%)	1(1.1%)	1.0
Herpes zoster	2(2.2%)	0	2(2.2%)	0.49

Overall, 46% in IV Group and 54% in IM Group reported to have some degree of complication. but they were all mild, bearable and treatment was continued satisfactorily. One bothersome side effect was pain at injection site which was significantly more in the IM than IV group (p<0.001). Group B patients had low treatment acceptability due to significant pain in injection site. Elevation in ALT was less than three folds not requiring treatment interruption. Similarly, some patients had eosinophilia and complete workup was done to rule out other causes, yielding negative results. Mild ECG changes mainly T-wave inversion was also observed as a transient side effect during treatment.

**Fig.1:**
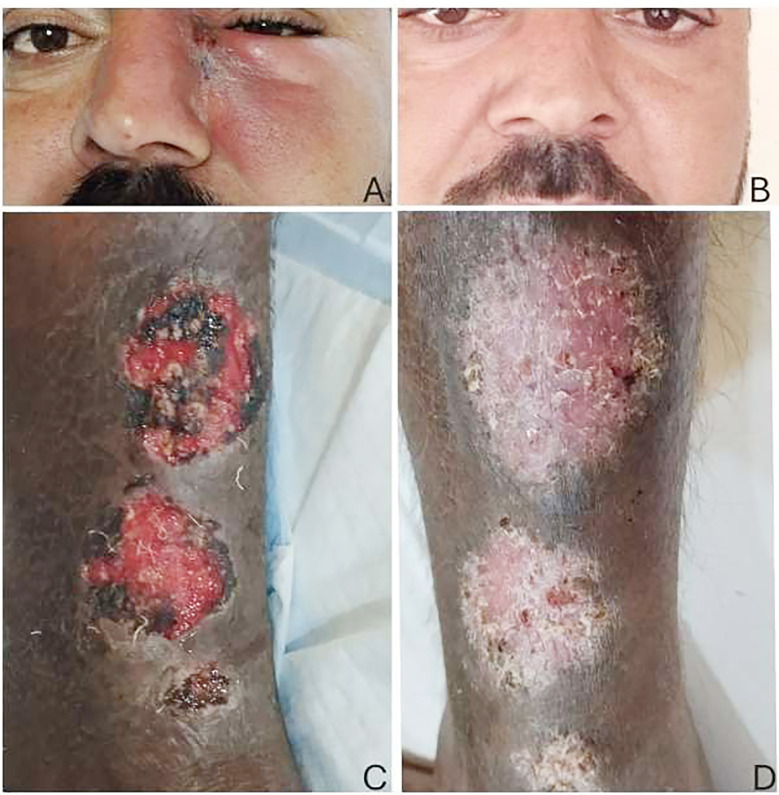
Cutaneous leishmaniasis (CL) treatment outcomes Group A. A: Facial lesions before treatment. B: Outcome after intravenous meglumine antimoniate (MA) treatment. C: CL lesions on the extensor aspect of the left leg before treatment. D: Post treatment outcome on the leg. Pictures shared with permission from patient.

**Fig.2:**
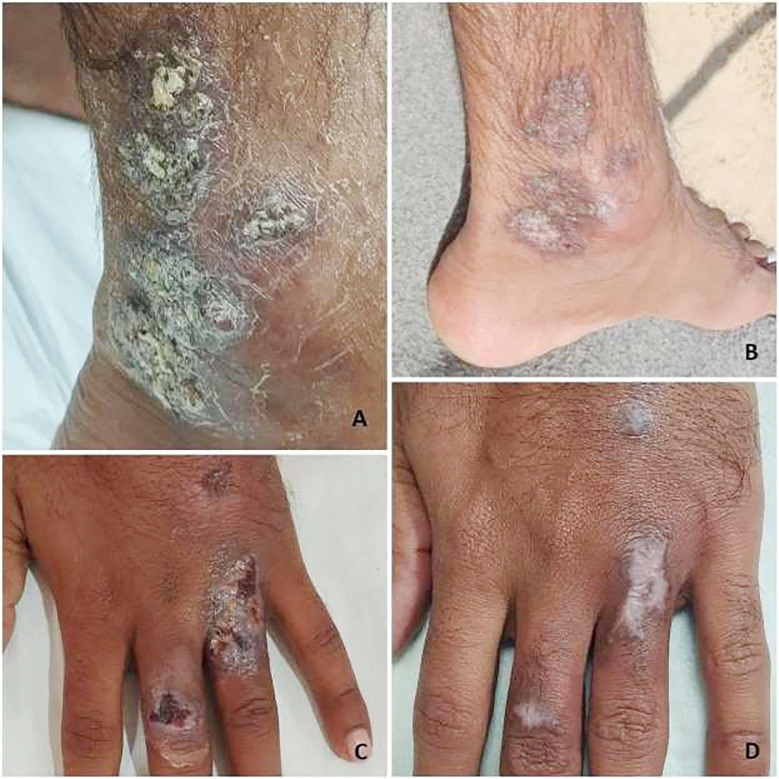
Cutaneous leishmaniasis (CL) treatment outcomes Group B. A and C are the lesions before treatment. B and D shows treatment response after 45 days. Pictures shared with permission from patient.

## DISCUSSION

Systemic antimonial have been used in patients of CL since 1929.^13^ In our study all participants were male which is consistent with the study done by Ahmed et al.^3^ Cloots et al.^14^ attributed this gender disparity to difference in traditional role of male and female in our society such as the nature of job. The average number of lesions in our IV group (4.78±3.87) was higher than the lesions 1.0 (1-2.5) in study done by Rodrigues.^15^ However, the number of lesions in our IM group (4.13±3.48) was similar to that observed in study by Munir et al.^16^ This observed increased number of lesions may be attributed to more outdoor activities, the higher number of insect bites seen in our population and also amount of inoculum injected with each bite.

IL and IM MA therapy have been compared in numerous studies. Lyra et al. stated IL MA was more efficient and safer than other parenteral therapies.^17^ Similarly, analysis by Brito et al. showed that IL treatment was more cost effective than the IV MA treatment.^18^ This can be due to the fact that IL provide high drug concentration within the lesion with less amount of drug wastage and can be done on outdoor basis, reducing hospital expenses. But there is scarcity of data directly comparing IM and IV routes. Rodrigues et al found 51(98%) cure rate with IV MA, three months after treatment.^15^

We had complete recovery 100% in our study after a mean 29.11±2.77 days in IV group. There was 60-90% cure rate of MA in study by Carvalho et al.^19^ while 34(94.4%) cure rate was reported by Saheki et al, at day 20 of IV MA.^11^ We had 38(84.4%) response at day 30. The possible explanation of the difference in results and slight slow response in our population may be due to that fact that above mentioned studies on IV MA were conducted on New World CL and there is insufficient data on effectiveness of IV MA in Old World CL. Also, there is difference in demographics of two population and the endemicity of different leishmania species in these two geographical areas.

Cure rate varies with route of drug delivery and treatment duration. In study of Khyber Pakhtunkhwa by Munir et al, there is 50% efficacy of IM MA at day 20 of treatment.^16^ Similarly, there is, 32(78%) efficacy of IM MA at week 4 (day 28) of treatment in a study by Paracha et al.^12^ Contrary to this, our study showed complete response in IM Group after an average time period of 39.56±2.80, roughly equivalent to 40 days. Greater time required to achieve desired results could be due to difference in route of drug administration, demographics or different definition of cure, though further research is needed in this area. Tareen et al demonstrated efficacy of 25(83.3%) with IM MA at day 21 to 28 of treatment. While In our study, there was 37(82.2%) cure rate at day 45 of treatment.^20^ We observed that healing in Group-B was slower compared to Group-A, which can be possibly due to appearance of resistance among different strains of leishmaniasis and drug susceptibility of parasite as proposed by Fernandez et al.^21^

Systemic toxicity of pentavalent antimonials is a major concern in treatment of CL.^22^ In a retrospective cohort study, Rodrigues et al. reported side effects in 28(53.80%) of patients from IV MA.^15^ Similarly, in our study among 45 group A patients 50.4% patients had adverse reaction. We observed comparable rates of arthralgia, 10(10.8%) vs. 7(13.46%); lower rate of myalgia 12(13.2%) vs. 11(21.15%), higher incidence of cardiotoxicity 11(24.4%) vs. 4 (7.69%) and headache 7(15.6%) vs. 4(7.69%) in our study compared to Rodrigue et al.^15^ These differences may be influenced by variation in sample size, patient population, or treatment protocols but all of them were manageable and none required treatment interruption. In contrast to Kaya et al.^23^ findings of significant decreases in neutrophil, lymphocyte and leukocyte counts, along with increases in liver enzymes (ALT, AST) and pancreatic enzymes, our study observed a significant elevation in eosinophil count, while the increase in ALT was not statistically significant. Barros et al.^24^ in his case report, also stated increased VZV reactivation during or immediately following treatment with antimony.

Time to resolution was different, 38(84.4%) in IV group versus 2(2.2%) in IM group at day 30. IV group patients healed earlier, which can be attributed to high plasma concentration of drug achieved after IV administration in contrast to variable absorption of drug when given via IM route. We also observed comparable side effects in both groups but there was significant difference in pain at injection site p< 0.05, which greatly affected patient’s compliance and confidence.

This study is unique in a way that we were not able to find a single study on comparison of parenteral routes of injection MA.

### Strengths of the study:

The strength of our study that it is directly applicable to clinical practice, provide insights into efficacy, safety, adverse effects and patient tolerance.

### Limitations:

It is a single-Centre study of short duration and small sample size due to which we cannot comment on long term implications and relapse after therapy. It is also possible that treatment response differs with sensitivity of different species to injection MA.

## CONCLUSION

Cutaneous leishmaniasis was effectively treated by both parenteral meglumine antimoniate but the intravenous route was found to be more effective and safer than the intramuscular route in the treatment of cutaneous leishmaniasis.

### Recommendations:

We found IV route to be more effective than IM route but further research is needed in this domain in terms of pharmacokinetics and pharmacodynamics of IV and IM administration. Data on drug use in special population such as children, elderly and those with co-morbidities is deficient, Patient preference, cost of each treatment modality and effect on patients quality of life can also be studied.
